# A study of dyslipidaemia drug prescribing pattern and cost of treatment in outpatient setting between Malaysian public hospitals and a teaching hospital

**DOI:** 10.1186/1471-2458-14-S1-O8

**Published:** 2014-01-29

**Authors:** Sit Wai Lee

**Affiliations:** 1 United Nations University International Institute for Global Health (UNU-IIGH), 56000 Cheras, Kuala Lumpur, Malaysia

## Background

In Malaysia, cardiovascular disease is the leading cause of death. Dyslipidaemia or high cholesterol level has been identified as one of the main risk factor that causes cardiovascular disease. In line with this, a clinical practice guideline on management of dyslipidaemia has been developed in 2004 and reviewed in 2011 for the management of dyslipidaemia cases in Malaysia. Drugs included in the management of dyslipidaemia are HMG CoA reductase inhibitors (statins), fibric-acid derivatives (fibrates), bile-acid sequestrants (resins), nicotinic acid (niacin) and its derivatives. The objective of the study was to evaluate the drug prescribing pattern in outpatient setting between Malaysian public hospitals and teaching hospital. The specific objectives of the study include the assessment of drug utilisation pattern for the treatment of dyslipidaemia, the assessment of cost of treatment for dyslipidaemia in the outpatient environment of Malaysian public hospitals and a teaching hospital and to relate the drug utilisation pattern of dyslipidaemia with the clinical practice guideline on management of dyslipidaemia.

## Materials and methods

This is a cross-sectional retrospective study with universal sampling of one year prescription data. Prescriptions will be collected at the selected public hospitals under Ministry of Health and a teaching hospital under Ministry of Education with electronic prescribing system in outpatient pharmacy department from 1 January 2012 to 31 December 2012. Prescription containing at least one dyslipidaemia drug is systematically sampled and evaluated. The cost of the dyslipidaemia drugs will be derived from the label purchased price of the drugs in outpatient. The data will be analysed and appropriate test will be carried to compare the two types of hospitals.

## Results

It is hope that this study will provide a trend of dyslipidaemia drug cost and utilisation in Malaysian public hospitals and teaching hospital.

## Conclusions

This study is important for the utilisation and cost of drugs used in the management of dyslipidaemia cases. This study will provide baseline data for the estimation of the economic implication of treating dyslipidaemia from the perspective of providers.

